# The impact of cell density variations on nanoparticle uptake across bioprinted A549 gradients

**DOI:** 10.3389/fbioe.2025.1584635

**Published:** 2025-04-30

**Authors:** Luigi Di Stolfo, Wang Sik Lee, Dimitri Vanhecke, Sandor Balog, Patricia Taladriz-Blanco, Alke Petri-Fink, Barbara Rothen-Rutishauser

**Affiliations:** ^1^ Adolphe Merkle Institute and National Center of Competence in Research Bio-Inspired Materials, University of Fribourg, Fribourg, Switzerland; ^2^ Department of Chemistry, University of Fribourg, Fribourg, Switzerland

**Keywords:** cell density, bioprinter, cell density gradients, SiO_2_ nanoparticles, nanoparticle uptake, advanced *in-vitro* model, nanoparticle-cell membrane interaction, 3D image analysis

## Abstract

**Introduction:**

The safe-by-design of engineered nanoparticles (NPs) for any application requires a detailed understanding of how the particles interact with single cells. Most studies are based on two-dimensional, uniformly dense cell cultures, which do not represent the diverse and inhomogeneous cell environments found *in situ*. *In-vitro* models that accurately represent tissue complexity, including realistic cell densities, are essential to increase the predictive accuracy of studies on cell-NP interactions. This study uses a bioprinted cell gradient model to examine the relation between cell density and NP uptake in one dish.

**Method:**

A549 lung epithelial cell density gradients within single inserts were produced with a bioprinter by modulating inter-droplet distances. After two days in culture, cells were exposed to Cy5-labeled silica NPs (SiO_2_ NPs, ∼112 nm, 20 μg/mL) for up to 48 h. Confocal fluorescence microscopy and 3D image analysis were used to quantify NP uptake, cell surface area, and cell volume. The relationship between NP uptake and the other parameters was then investigated statistically.

**Results:**

Bioprinting enabled the creation of reproducible linear cell density gradients, allowing controlled modeling of density variations while preserving cell viability throughout the experiment. Increasing inter-droplet distances, from 0.1 mm to 0.6 mm, were used to achieve uniformly decreasing cell densities. SiO_2_ NP uptake per cell was around 50% higher in low-density regions compared to high-density areas across all time points, i.e., 6, 24, and 48 h post-exposure. This inverse relationship correlated with greater average cell surface area in lower-density regions, while differences in the proliferation rates of the A549 cells at varying densities did not significantly impact uptake, did not significantly impact uptake.

**Conclusion:**

SiO_2_ NP uptake is significantly enhanced at lower cell densities, mainly due to the increased available surface area, revealing potential cell-NP interaction differences in tissues that present cell density variability. Our drop-on-demand bioprinting gradient model successfully supports the implementation of cell density gradients in *in-vitro* models to increase their relevance as new approach methodologies (NAMs) for next-generation risk assessment strategies.

## 1 Introduction

Gradients, variations of a particular variable over a given distance or over time, are a recurring feature in nature and are present in all biological contexts, from simple monocellular life forms to complex macroscopic organisms (Benyamin et al., 2023; Jo et al., 2022; Bernhardt et al., 2022). These varying parameters include biomolecule and chemical concentrations (Majumdar et al., 2014; DeRocher et al., 2020), electrical potential (McCaig et al., 2009; Adams and Levin, 2013), and mechanical stiffness (Shellard and Mayor, 2021; Oudin and Weaver, 2016) but are not limited to these factors. In human biology, among the various kinds of gradients, cell density variations are crucial in both physiological and pathological conditions (Cammarota et al., 2024; Zheng et al., 2016; Trajkovic et al., 2019; Greene et al., 2016; Sarate et al., 2024; Lu et al., 2024). *In-vivo*, fluctuations of cell density over time contribute to processes like tissue repair, development, and aging, while being driven by immune infiltration, epithelial remodeling, regeneration, and morphogenesis (Cammarota et al., 2024; Zheng et al., 2016; Sarate et al., 2024; Lu et al., 2024; Klohs et al., 2008).

Despite the critical and not yet fully understood impact of cell density as a research parameter, it is often primarily considered in studies focused on cell proliferation, differentiation, or migration (Enrico Bena et al., 2021; Zhou et al., 2011; Wroblewski et al., 2022; Heinrich et al., 2020; Devany et al., 2023; Burger et al., 2022), and overlooked in NP hazard and cell interaction studies. Cell density gradients and inhomogeneities are particularly interesting in NP applications because the way NPs interact with cells, their uptake efficiency, and intercellular trafficking can change depending on how densely and spatially packed the cells are (Greene et al., 2016; Zhang et al., 2021; Garcia Romeu et al., 2021; Yacobi et al., 2008). Notably, cell density impacts the observed magnitude and kinetics of adverse effects by modulating cellular responses to external agents, such as drugs and oxidative stress-inducing compounds (Riss and Moravec, 2004; Caviglia et al., 2015; Wu YK. et al., 2020). Since these effects depend on cellular interaction and uptake, similar effects could influence NP delivery efficiency and the resulting toxicological responses. Due to these density-dependent changes, the reliability and reproducibility of experimental results, particularly *in-vitro* studies, may be affected (Trajkovic et al., 2019; Greene et al., 2016).

A previous research study (Kim et al., 2012) has investigated how cell cycle phase influences NP uptake and suggested that factors such as cell density may indirectly affect internalization through their impact on cell cycle dynamics. A higher degree of confluency in *in-vitro* cultures has been shown to increase the proportion of cells in the G0/G1 phase (Hayes et al., 2005; Choresca et al., 2009), which, as demonstrated in the referenced study, is associated with reduced NP uptake. Most 2D *in-vitro* models only assume a single cell density at the time of the experiment, and, in addition, this parameter is often unreported. Furthermore, conventional pipetting for cell seeding can produce uneven distributions (Moore et al., 2019), as turbulent flow, convection, and vessel movement cause cells to aggregate at the center or edges of the dishes/wells. In smaller wells (e.g., 96- to 24-well formats), the meniscus becomes an additional factor that affects the cell distribution and the experimental results (Reynolds et al., 2018; Glaubitz et al., 2023; Mansoury et al., 2021). While these effects may replicate aspects of *in-vivo* heterogeneity, their random and uncontrolled nature prevents systematic investigation and introduces variability, resulting in limited reproducibility.

To address these challenges and improve both the reproducibility of *in-vitro* studies and their ability to better mimic relevant *in-vivo* conditions, various methods have been developed to generate controlled cell density gradients, each with specific advantages and limitations. These methods include microfluidic platforms that enable precise control of gradients through fluid flow (Mehling and Tay, 2014; Chiu et al., 2000; Li et al., 2015a), directed cell migration systems that generate gradients through mechanical, chemical, and electrical factors (Mao et al., 2016; Smith et al., 2006; Liu et al., 2007; Wan et al., 2009), sedimentation-based approaches utilizing tilted surfaces to create cell concentration gradients (Li et al., 2015a; Liu et al., 2013), and 3D bioprinting techniques that allow controlled deposition of cell-laden bio-inks (Kuzucu et al., 2021; Idaszek et al., 2019). However, among these approaches, only a few have demonstrated feasibility for creating cell density gradients (Chiu et al., 2000; Li et al., 2015a; Liu et al., 2013; Kuzucu et al., 2021).

Developing a straightforward, reproducible, and efficient method to create controlled cell density gradients in one cell culture dish would address the standardization issues in current methods and the need for more physiologically relevant *in-vitro* cell culture models to study the effects of NPs. This study examines the connection between human lung epithelial cell density and NP-cell interactions by developing a reproducible gradient method using bioprinting drop-on-demand technology and investigating the relationships between cell density, proliferation rate, surface area, and NP uptake. The findings demonstrate the reproducibility of the cell gradient after optimization of the bioprinting parameters without impacting cell viability. Most significantly, a strong negative correlation between NP uptake per cell and cell density was shown, providing critical information to understand the broader impact of cell density on NP exposure, particularly in pathological scenarios.

## 2 Materials and methods

### 2.1 Cell culture

Cell culture reagents were sourced from Gibco, Thermo Fisher Scientific (Zug, Switzerland) unless stated otherwise. The A549 cell line was obtained from the American Type Culture Collection (ATCC, Rockville, MD, USA) and grown in RPMI-1640 medium. This medium was supplemented with 10% fetal bovine serum (FBS), 2 mM L-Glutamine, and Penicillin-Streptomycin (100 μg/mL). This complete growth medium is referred to as complete RPMI 1640 (cRPMI). Cell cultures were kept at 37°C in an incubator with 5% CO_2_ and 95% humidity.

A549 cells were split twice a week using 0.25% Trypsin-EDTA, following the ATCC guidelines. Cell concentration was checked using the trypan blue exclusion method (0.4% trypan blue in phosphate-buffered saline (PBS), pH 7.2, from Gibco, Life Technologies Europe B.V., Zug, Switzerland), and counted with an automated cell counter (EVE, NanoEnTek Inc., Seoul, South Korea). The cells were maintained at 37°C with 5% CO_2_ and 95% humidity and passaged when they reached 80%–90% confluency, with internal passage numbers ranging from 6 to 18. Regular *mycoplasma* testing was performed, and no contamination was observed at any time.

### 2.2 Fabrication of bioprinted cell gradients

The printing instrument used in this study was the 3DDiscovery™ Biosafety Bioprinter from regenHU Ltd., Villaz-Saint-Pierre, Switzerland. Dispensing is performed using CF300N valve-based print heads, which support jetting or contact dispensing through a solenoid-actuated micro-valve. A549 cell suspensions were pipetted into cartridges connected to the print heads via a Luer-Lock adapter and agitated using a propeller to avoid sedimentation. Dispensing is driven by filtered air pressure. Designs were created in BioCAD (version 1.1), converted into g-code, and executed following a parameter adjustment for feed rate, valve opening time, inter-droplet distance, and air pressure.

In this article, the term “pattern” refers to the result of the droplet-by-droplet cell deposition process. “Graded patterns” or simply “cell density gradients” or “cell gradients” indicate the continuous cell density gradient models, while “control patterns” refer to the uniform-density models printed as separate controls for each density condition.

Graded patterns and uniform control patterns of A549 cells were fabricated using the aforementioned valve-based droplet deposition technology. Cells were deposited onto 3 µm holey polyethylene terephthalate (PET) hanging inserts (Corning^®^ Falcon, Reinach, Switzerland) placed in 6-well plates, each with a membrane surface area of 4.2 cm^2^.

The print parameters included a valve opening time of 150 µs, an air pressure of ∼30 kPa, a feed rate of 10 mm/s, and a 0.2 mm nozzle diameter. Cell suspensions had a concentration of 2 × 10^6^ cells/mL. Prior to the deposition of the cells, 1.5 mL of complete RPMI was added to each well, and PET inserts were placed inside the well. Then, a preliminary gradient of only the culture medium was deposited onto the PET inserts. This initial medium layer facilitated consistent merging between subsequent cell-laden droplets and was designed with an opposing spacing pattern to the following cell printing process.

To create continuous cell density gradients, the spacing between printed cell-containing droplets was systematically varied. Starting with closely spaced droplets (0.1 mm apart) at one end, we gradually increased this spacing by 0.05 mm increments every 4 mm of print distance. This resulted in a continuous gradient with maximum droplet spacing (0.6 mm) at the opposite end. For control patterns, presenting different cell densities on distinct inserts, inter-droplet distances of 0.125 mm, 0.35 mm, and 0.575 mm were used to achieve high, medium, and low cell densities, respectively. In these control patterns, the inter-droplet distances were slightly adjusted to account for the gradual blending of density steps observed in the gradient patterns. After printing, 0.5 mL of complete RPMI medium was added to the lower compartment of each well plate to reach 2 mL, while no additional medium was added to the top of the inserts. This ensured that cells could grow within the defined “linear cell medium zone” established during printing. Cells were then incubated for 2 days before further experiments were performed. Subsequent imaging and staining procedures are described in detail in Section 2.4.

### 2.3 Synthesis and characterization of silica (SiO_2_) NPs

Fluorescent SiO_2_ NPs incorporating Cy5 dye were synthesized via the Stöber method with slight modifications (Stöber et al., 1968; Moreno-Echeverri et al., 2022). Initially, 162 mL of absolute ethanol (EtOH) (VWR, Dietikon, Switzerland), 58 mL of Milli-Q water, and 8.71 mL of 25% ammonium hydroxide (NH_4_OH, Merck, Zug, Switzerland) were mixed in a 500 mL round-bottom flask equipped with a reflux setup. The mixture was heated to 70°C under constant magnetic stirring for 30 min. Then, 22 mL of tetraethyl orthosilicate (TEOS, GC grade, ≥99.0%, Si(OC_2_H_5_)_4_, Sigma-Aldrich, St. Louis, MO, USA) was quickly introduced. After 2 min, 200 µL of a solution containing 11.5 mg/mL Cyanine 5 N-Hydroxysuccinimide ester (Cy5-NHS, Lumiprobe, Hunt Valley, MD, USA) in dimethyl sulfoxide (DMSO, GC grade, ≥99.9%, Sigma-Aldrich, St. Louis, MO, USA), and 3 µL of (3-aminopropyl) triethoxysilane (APTES, H_2_N(CH_2_)_3_Si(OC_2_H_5_)_3_, GC grade, 99%, Sigma-Aldrich, St. Louis, MO, USA) were added to the flask. The reaction was continued at 70°C for an additional 4 h. Upon completion, the dispersion was cooled down to room temperature and centrifuged at 3,000 rpm for 40 min three times. The NPs were re-dispersed in Milli-Q water and stored in the dark at 4°C for up to 1 month. The NP stability in complete RPMI was analyzed by DLS and TEM over 6, 24, and 48 h. Moreover, the stock NPs in Milli-Q water were analyzed by TEM before and after all uptake experiments to ensure no degradation occurred during storage (Supplementary Figure S3).

The SiO_2_ NPs were imaged using transmission electron microscopy (TEM; FEI Technai G2 Spirit, Columbia, MD, USA), and their core diameter along with size distribution were analyzed using ImageJ software (Wayne Rasband, National Institute of Health, Bethesda, MD, USA). The hydrodynamic diameter and zeta potential of the NPs were determined using an Anton Paar Litesizer 500 particle analyzer (Anton Paar, Graz, Austria) with a 658 nm laser by dynamic light scattering (DLS). The hydrodynamic size was obtained at a scattering angle of 175° and using an advanced cumulant model. Applying the Smoluchowski approximation yielded the zeta potential. The hydrodynamic diameter was measured at 25°C in Milli-Q water and complete RPMI after 0, 6, 24, and 48 h incubation times at 37°C. The incubated NPs were then centrifuged (Thermo Scientific Heraeus Multifuge X1R Pro Centrifuge) at 10,000 rpm for 15 min, and the resulting supernatant was collected for analysis to assess the loss of fluorescent labels from the NPs. Liquid-phase fluorescence spectra were recorded using a Horiba Fluorolog 3 spectrometer (Horiba, Kyoto, Japan), equipped with a 450 W Xenon lamp for excitation and an FL-1030-UP photomultiplier for detection. The excitation wavelength was set to 650 nm, and fluorescence intensity was measured from 650 nm to 800 nm.

### 2.4 Confocal microscopy imaging

After exposure of the cells to 1 mL 20 μg/mL SiO_2_ NP dispersion in complete RPMI (see Section 2.3) for the three time points of interest (i.e., 6, 24, and 48 h), the inserts were washed three times with PBS and then fixed with a 4% paraformaldehyde (PFA) (Sigma-Aldrich, St. Louis, MO, USA) solution in PBS at +4 °C for at least 60 min. Following fixation, PBS washing, and permeabilization with 0.1% Triton X-100 (Thermo Fisher Scientific, Switzerland) for 10 min, cells were stained for 60 min at room temperature. Both cell nuclei and F-actin filaments were stained simultaneously using a single solution containing 1 μg/mL 4′,6-diamidino-2-phenylindole (DAPI), and 66 nM Alexa Fluor 488 phalloidin (Thermo Fisher Scientific, Switzerland) in PBS. After staining, cells were washed three times with PBS. Coverslips were then mounted with Kaiser’s glycerol gelatin and stored in the dark at 4°C.

Imaging was conducted using a Leica Stellaris 5 inverted confocal laser scanning microscope (cLSM, Leica Wetzlar, Germany) equipped with Power HyD S detectors, a Plan FLUOTAR 10x/0.32 Dry objective, a Plan-Apochromat 20x/0.75 Dry objective, and a Plan-Apochromat 63x/1.4 Oil CS2 objective (Leica, Wetzlar, Germany). The system was operated using LAS X software version 4.6.1. Stack images of the cells were acquired sequentially at ×63 magnification, providing a field of view of 184.70 µm × 184.70 µm with a pixel resolution of 1,024 × 1,024. For broader overviews, tile scans across full graded patterns were performed using ×10 magnification, while tile scans of the control patterns for cell counting were obtained using 20x magnification. The resulting tiles were merged using the LAS X software. Fluorescence imaging was performed using three laser excitation wavelengths: 405 nm for DAPI, 488 nm for Alexa Fluor 488, and 638 nm for Cyanine 5 (Cy5).

### 2.5 3D image analysis

3D Stack images of the cells were acquired and analyzed in three dimensions using Imaris software (Oxford Instruments, Version 10.1, Abingdon, UK). Initially, all image stacks were trimmed at the level of the insert membrane to establish a consistent starting point for subsequent analysis across samples. The Imaris “Surfaces” function created a three-dimensional region of interest (ROI) by training a model to distinguish the cellular mass from surrounding external space (Supplementary Figure S4). This ROI allowed the assessment of several parameters, including NP uptake and average surface area and volume.

NP uptake was quantified by integrating the signal of Cy5-conjugated NPs throughout the entire cell volume within the defined ROI. Additionally, the accessible surface area and total volume were calculated directly from the dimensions of the ROI itself.

The “Spots” function of Imaris was then used to determine the number of cell nuclei per field of view (Supplementary Figure S4). The number of nuclei was accurately identified by inputting an estimated nucleus diameter (9 µm in X-Y, 6 µm in Z) and applying a background subtraction filter. All data obtained from these analyses were subsequently used for statistical evaluation.

### 2.6 Cytotoxicity, viability, and proliferation assays

To evaluate the impact of the printing process and NP exposure on cell viability within the printed graded patterns, the ReadyProbes^®^ Cell Viability Imaging Kit (Blue/Green) (Thermo Fisher Scientific, Waltham, MA, USA) was used following the standard protocol. The test cell gradients were printed and incubated for 48 h, before ulterior 48 h exposure to the NPs. Fluorescence imaging was performed to assess cell integrity, staining all nuclei with NucBlue^®^ Live reagent (405 nm excitation), a cell-permeable nuclear stain based on Hoechst 33342, and selectively staining dead cells with NucGreen^®^ Dead reagent (488 nm excitation), a membrane-impermeant DNA dye.

For cell viability control experiments, the different densities of the graded patterns were reproduced in separate wells. The cells were seeded in a 96-well plate at varying densities (Table 1) and allowed to grow until exposed to 100 µL of SiO_2_ NPs (20 μg/mL) for the three chosen time points. The seeding densities were adjusted to ensure that cells reached the three conditions of interest—high (600 cells/mm^2^), medium (300 cells/mm^2^), and low (150 cells/mm^2^)—at the moment of NP exposure, which occurred in succession at 48, 24, and 6 h before the assay endpoint. To design the experimental setup, a constant 24 h doubling time was assumed based on reported values for A549 cells (Assanga, 2013; American Type Culture Collection, 2025), allowing synchronization of all conditions within a single assay run. This minimized the variability induced by running viability assays on different days for each different condition. In summary, after 24 h of pre-incubation, the NPs were added sequentially, allowing all cells to reach their respective exposure endpoints simultaneously after 48 h.

**TABLE 1:** Initial Cell Seeding Densities Optimized for Simultaneous NP Exposure Endpoints (cells/mm^2^). Table 1 presents optimized initial cell seeding densities for cytotoxicity assays with varying NP exposure durations. We established these seeding parameters to ensure cells reached comparable densities. We established these seeding parameters to ensure cells reached comparable densities at the time of NP exposure, regardless of the exposure duration. This standardization allows for a direct comparison of cytotoxic effects across different exposure time points. For example, cells intended for 6 h NP exposure experiments were initially seeded at ∼28 cells/mm^2^ and pre-incubated for 66h, reaching ∼150 cells/mm^2^ before NP addition. Similarly, for 48 h exposure experiments, cells were seeded at a higher density (∼75 cells/mm^2^) and pre-incubated for only 24 h to achieve the same cell density at exposure time. This approach eliminates potential confounding effects of varying cell confluence during cytotoxicity assessment, enabling simultaneous endpoint analysis at 72 h post-initial seeding across all experimental conditions.

Time points	Cell density (cells/mm^2^)		
	Low	Medium	High
6 h	∼28	∼55	∼110
24 h	∼38	∼75	∼150
48 h	∼75	∼150	∼300

After exposure, the conditioned medium was transferred to a second 96-well plate, maintaining the same layout. 100 μL of fresh complete RPMI was added to the cells, and Water Soluble Tetrazolium-1 (WST-1) (Roche, Mannheim, Germany) and Lactate Dehydrogenase (LDH) (Roche, Mannheim, Germany) assays were conducted to assess cellular metabolic activity and membrane integrity.

As a positive control, 0.2% Triton X-100 in complete RPMI was added to the cells to ensure full lysis before the supernatant was collected. LDH and WST-1 levels were measured in triplicate. The absorbance of the colorimetric product was read using a spectrophotometer (Benchmark Microplate reader, BioRad, Cressier, Switzerland) at 490 nm and a reference wavelength of 630 nm for LDH and at 440 nm with a 650 nm wavelength as reference for WST-1. Interference was accounted for by using appropriate blanks to eliminate any potential quenching or auto-absorption effects. The absorbance results for the LDH and WST-1 assays were normalized to the positive control (0.2% Triton X-100) and the untreated negative control, respectively.

To address the possible impact of NP exposure on proliferation, cells were seeded at different densities in a 24-well plate and exposed to 500 µl of NP dispersion (20 μg/mL). Initial seeding densities were 300 cells/mm^2^, 150 cells/mm^2^, and 75 cells/mm^2^, representing high, medium, and low densities, respectively, and were intended to double after 1 day of pre-incubation. After NP exposure for three time intervals (6, 24, and 48 h), the cells were washed with PBS and detached using Trypsin/EDTA. Following detachment, cells were collected with complete RPMI and subsequently counted using an automated cell counter (EVE, NanoEnTek Inc., Seoul, South Korea) to determine proliferation rates with and without NPs.

The abovementioned target cell densities for cytotoxicity and proliferation assays were selected based on preliminary experiments, which revealed significantly higher proliferation rates in well plates compared to the printed setup. This prompted a reduction in initial seeding densities to account for the faster proliferation. As a result, at the 6 h exposure point, well plate densities were lower than those in printed control patterns. However, by 24 and 48 h of exposure, their rapid proliferation led to comparable or even higher densities than those observed in the control patterns (Supplementary Figure S1).

### 2.7 Statistical analysis

Average NP uptake was calculated by dividing the total NP fluorescence intensity by the cell count per field of view. Similarly, average free surface area and cell volume were determined by dividing the cell clusters’ total surface area and total volume by the number of cells, defined by the number of nuclei, in each field of view. Data were collected from three independent samples per exposure time point, each comprising 15 confocal stacks—five stacks per density region (high, medium, low). Similarly, for the control patterns, data were collected from three samples (one per density condition) at each exposure time point. Group sizes were kept equal across all density regions to ensure consistency in statistical analysis.

Statistical analysis was performed using GraphPad Prism version 10.4.1 for Windows (GraphPad Software, www.graphpad.com). Raw “NP uptake per cell” (Cy5 signal intensity, A.U.) data was organized in different columns for each density region and further separated into subcolumns by biological repetition. Two-way ANOVA followed by Tukey’s multiple comparisons test was conducted to analyze the data. Two-way ANOVA was selected over one-way ANOVA to account for biological repetition as a matched factor, allowing for a more precise comparison of treatment effects while controlling for within-replicate variability.

Normality testing was carried out on all raw data using Shapiro-Wilk (ideal for small datasets like those used in this study), Anderson-Darling, Kolmogorov-Smirnov, and D'Agostino-Pearson tests. Raw data passed the Shapiro-Wilk test in all but one case, while the ANOVA residuals consistently followed normality. Homogeneity of variances was tested using the Levene-Brown-Forsythe test, which was successful in all cases except one, with a p-value of 0.0466. ANOVA is robust to slight deviations from homoscedasticity; therefore, the analysis was still considered valid. The Geisser-Greenhouse correction was applied to all datasets as a precautionary measure to account for potential minor sphericity deviations.

The statistical comparison of NP uptake (per cell) between different cell densities was additionally analyzed using a Mixed-Effects Model, which confirmed the trends initially observed with the ANOVA analysis without assuming the normality of residuals or homoscedasticity.

The same flow was used for the statistical analysis of NP uptake per unit of surface area (µm^2^) at each cell density, where the measured heterogeneity of variances justified the use of the Mixed-Effects Model instead of Two-way ANOVA.

Pearson’s and Spearman’s correlation tests were applied to determine the correlation between NP uptake, cell density, average free surface area, and cell volume, given that not all data sets were normally distributed. Proliferation data were analyzed and fitted using exponential (Malthusian) growth models to calculate doubling times.

## 3 Results

This study examined the interactions between SiO_2_ NPs and A549 cells at different cell density conditions, focusing on cell viability, density-dependent uptake profiles, and the effects of proliferation.

### 3.1 Effects of SiO_2_ NPs on cell growth and viability

First, the three densities were replicated in single wells to analyze the NP-cell interactions in individually controlled settings. Cells were seeded at varying concentrations (Table 1) in a 96-well plate and pre-incubated for 24 to 66 h prior to NP exposure. Seeding was adjusted to achieve high (600 cells/mm^2^), medium (300 cells/mm^2^), and low (150 cells/mm^2^) densities at the time of NP exposure.

**FIGURE 1:**
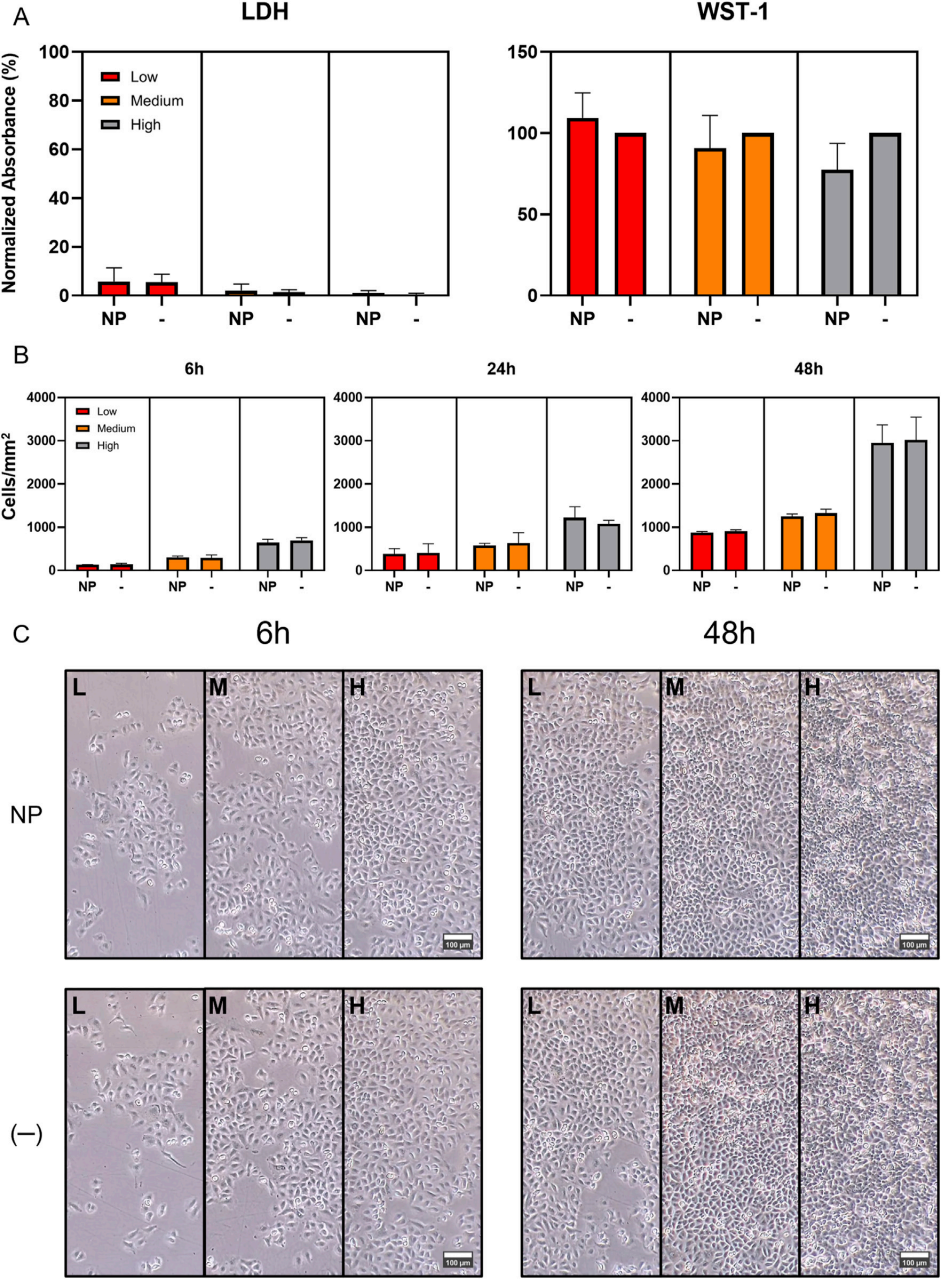
Impact of SiO_2_ Nanoparticles (SiO_2_ NPs) on A549 Cell Proliferation, Viability and Cytotoxicity. **(A)** LDH and WST-1 cytotoxicity and viability assays at 48 h exposure time point. The assays displayed expected outcomes, with the WST-1 results revealing a noticeable, but statistically non-significant reduction in metabolic activity at higher cell densities. This may be due to the assay’s mechanism, which relies on intracellular NADPH to reduce the WST-1 salt. Mitochondria are a primary NADPH source and can be subtly affected by SiO_2_ NPs. The effect may be more evident at higher cell densities due to higher baseline metabolic activity. Absorbance values were normalized to the 0.2% Triton X-100 positive control for LDH, and to the untreated negative control for WST-1. **(B)** Comparison of A549 cell proliferation with and without SiO_2_ NP exposure over 48 h revealed no significant impact of the NPs on cell doubling time. **(C)** Comparison of phase-contrast images of A549 cells, used in the proliferation investigation, from left to right: low, medium, and high initial densities, under control conditions (−) and with NP exposure (NP) at 6 and 48 h time points.

The SiO_2_ NPs were synthesized, and their size, zeta potential, and colloidal stability in complete RPMI were characterized. The results are summarized in Table 2. The cells were then sequentially treated with the NPs (20 μg/mL) at three different time points, ensuring all reached their final exposure endpoint simultaneously after 48 h. SiO_2_ NPs demonstrated minimal cytotoxicity across high, medium, and low cell densities over 6, 24, and 48 h (Figure 1). The LDH assay showed negligible impact on membrane integrity, while WST-1 measurements revealed only a slight, statistically non-significant reduction in metabolic activity, particularly at higher densities after 48 h (Figure 1A).

**TABLE 2:** Summary of the Key Physicochemical Properties and Stability of the SiO_2_ NPs. ^1^core diameter; ^2^hydrodynamic diameter; ^3^zeta potential.

TEM	DLS				
	MilliQ		cRPMI		
C_d_^1^ [nm]	H_d_^2^ [nm] t = 0 h	ζ^3^ [mV]	H_d_ [nm] t = 0 h	H_d_ [nm] t = 6 h	H_d_ [nm] t = 48 h
112 ± 14 nm	121 ± 2 nm	−47.5 ± 0.6 mV	160 ± 9 nm	152 ± 4 nm	147 ± 7 nm

To evaluate the impact of NP exposure on proliferation, the cells were seeded in a 24-well plate at high (300 cells/mm^2^), medium (150 cells/mm^2^), and low (75 cells/mm^2^) densities and pre-incubated for 24 h. They were then exposed to 500 µL of NP dispersion (20 μg/mL). Cell counts at 6, 24, and 48 h post-treatment showed stable proliferation rates across all densities (Supplementary Figure S1), with no significant differences due to NP exposure (Figure 1B). Furthermore, phase-contrast microscopy revealed no noticeable changes in cell morphology or growth patterns over the 48 h period (Figure 1C).

### 3.2 A549 cell gradient fabrication by bioprinting technology

**FIGURE 2:**
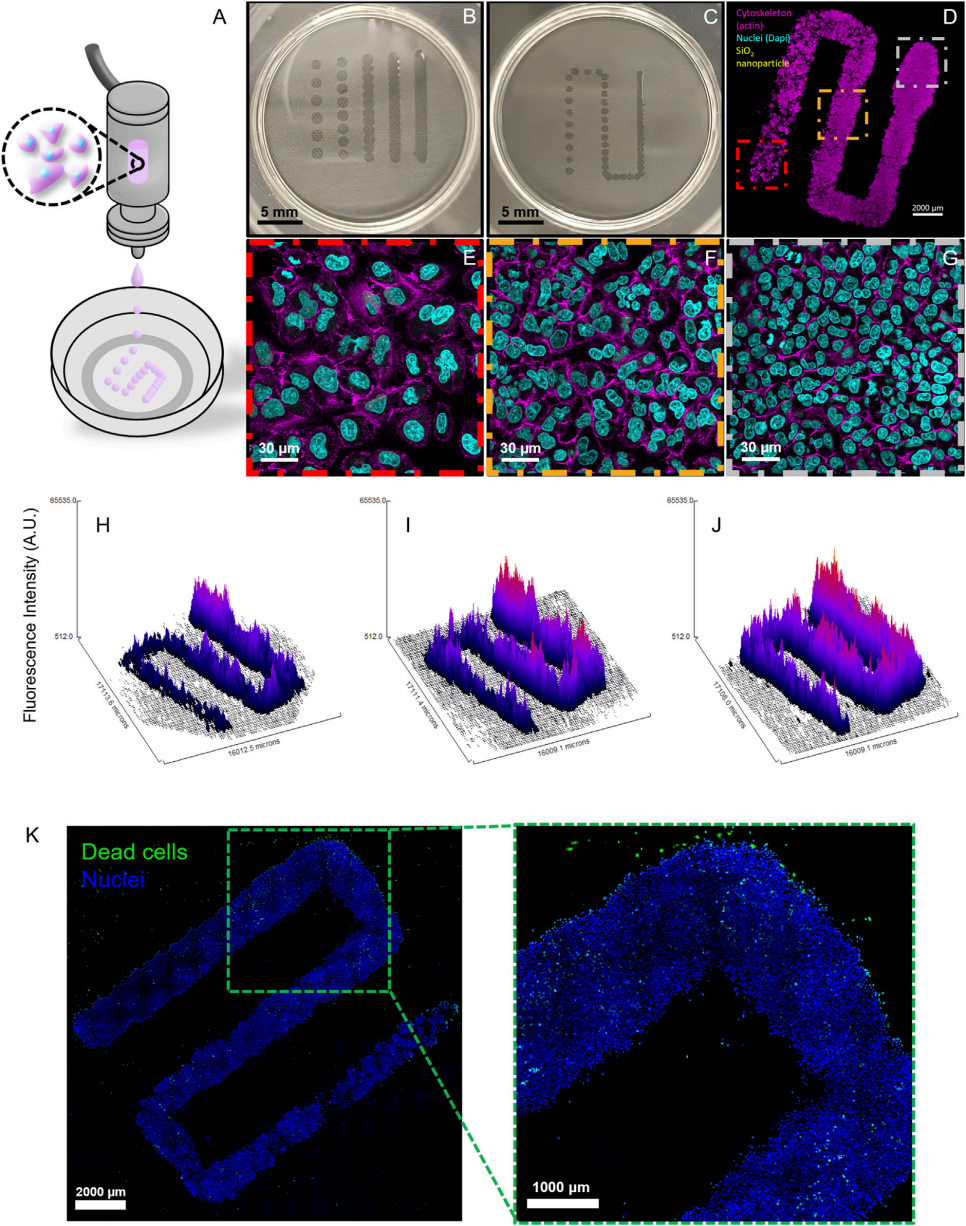
Printing Optimization and Cell Gradient Fabrication. **(A)** An illustration of the printing process shows a drop-on-demand printhead depositing cell-laden droplets with varying spacing. **(B)** PET culture inserts with printed lines at different fixed inter-droplet distances. **(C)** PET culture insert showing a graded deposition pattern with progressively increasing inter-droplet distances. **(D)** Confocal Laser Scanning Microscopy (cLSM) fluorescent image of the gradient after 4 days of incubation, demonstrating the established cell density gradient. **(E–G)** Close-up (63x) images from the three studied density zones of the gradient, highlighting variations in cell distribution and density. The printed cells were stained with Alexa Fluor 488 phalloidin (magenta) to visualize F-actin and with DAPI (cyan) to label cell nuclei. **(H–J)** Representative surface plots generated from 10x tile images of graded patterns at 6, 24, and 48 h time points from the actin channel, displaying actin fluorescence intensity as an indicator of cell density. **(K)** A cLSM 10× tile scan image of a gradient stained with the Live/Dead imaging kit (dead cells in green; nuclei in blue) reveals minimal cell death after printing, pre-incubation, and 48-h NP exposure.

The illustration in Figure 2A portrays the printing process where a drop-on-demand printhead deposits cell-laden droplets with a gradually increasing inter-droplet spacing (from an initial 0.1 mm, increasing by 0.05 mm every 4 mm until reaching 0.6 mm) to generate the cell density gradient on PET hanging inserts with 3 µm pores. The choice of these inserts as a printing substrate for lung tissue modeling stems from previous works of this group (Horváth et al., 2015). Direct printing on plastic or glass dishes, both clean and functionalized, was also attempted, but the droplets spread immediately, leading to less controlled conditions (data not shown). The printed cell patterns on the PET inserts maintained consistent structure and spacing on the membrane surface (Figure 2B), enabling the formation of defined cell density gradients (Figure 2C). After the printing, the inserts were placed into the 2-well chamber system with cell culture medium on the lower side in the basal compartment. Due to the stress from printing, the graded patterns were pre-incubated for 48 h, rather than 24 h, to allow cell recovery before NP exposure.

After this pre-incubation time, cell culture medium was added to the apical side by pipetting, and the system was incubated for an additional 48 h; this final incubation period represents the longest exposure time, simulating the conditions of pre-incubation and subsequent NP exposure. Fluorescence microscopy of stained cells and cell nuclei after incubation revealed distinct cell density regions across the gradient (Figure 2D), with clearly defined low (red box), medium (orange box), and high (gray box) density zones (Figure 2E–G).

Due to the inability to clearly define the boundaries of the three density zones within the printed graded patterns, wide-area density quantification was not feasible. Instead, control patterns were fabricated on three separate inserts using inter-droplet distances of 0.125 mm, 0.35 mm, and 0.575 mm to achieve high, medium, and low cell densities, respectively, providing reference values and serving as the basis for proliferation analysis (see Section 3.4; densities are presented in Table 3). For the graded patterns, cell densities were calculated from high-magnification (63x) image stacks and were used exclusively for direct correlation with NP uptake.

**TABLE 3:** *Average Cell Densities (Mean ± SD) Measured from Control Patterns for High, Medium, and Low Density Conditions at each Time Point (cells/mm^2^) *n counts = 3.

Time points	Low (0.575 mm)	Medium (0.35 mm)	High (0.125 mm)
6 h	272 ± 40	568 ± 163	1,069 ± 197
24 h	386 ± 67	947 ± 282	1,655 ± 195
48 h	821 ± 77	1,817 ± 352	2,655 ± 298

Surface plot analysis demonstrated the achievement of stable cell gradients with uniform proliferation dynamics over time (Figure 2H–J). From end to end, the gradient consisted of a continuous decrease in cell crowding, with reproducible cell densities across multiple fabrication runs in the three analyzed density zones. Specifically, after optimizing the printing parameters through several test runs, three independent printing processes (biological repetitions) were conducted, each using cells from a different passage. In addition, three graded patterns were produced per experiment, one for each exposure time point. All runs were performed by a single operator; however, the bioprinter’s automation minimizes potential inter-operator variability, as their role is limited to cartridge preparation and initiating the printing process via the user interface.

Live/dead imaging was used to assess the viability of the printed cells after a 48 h pre-incubation period followed by an additional 48 h of NP exposure, revealing minimal cell death (Figure 2K).

### 3.3 The impact of A549 cell density gradients on SiO_2_ NP uptake

**FIGURE 3:**
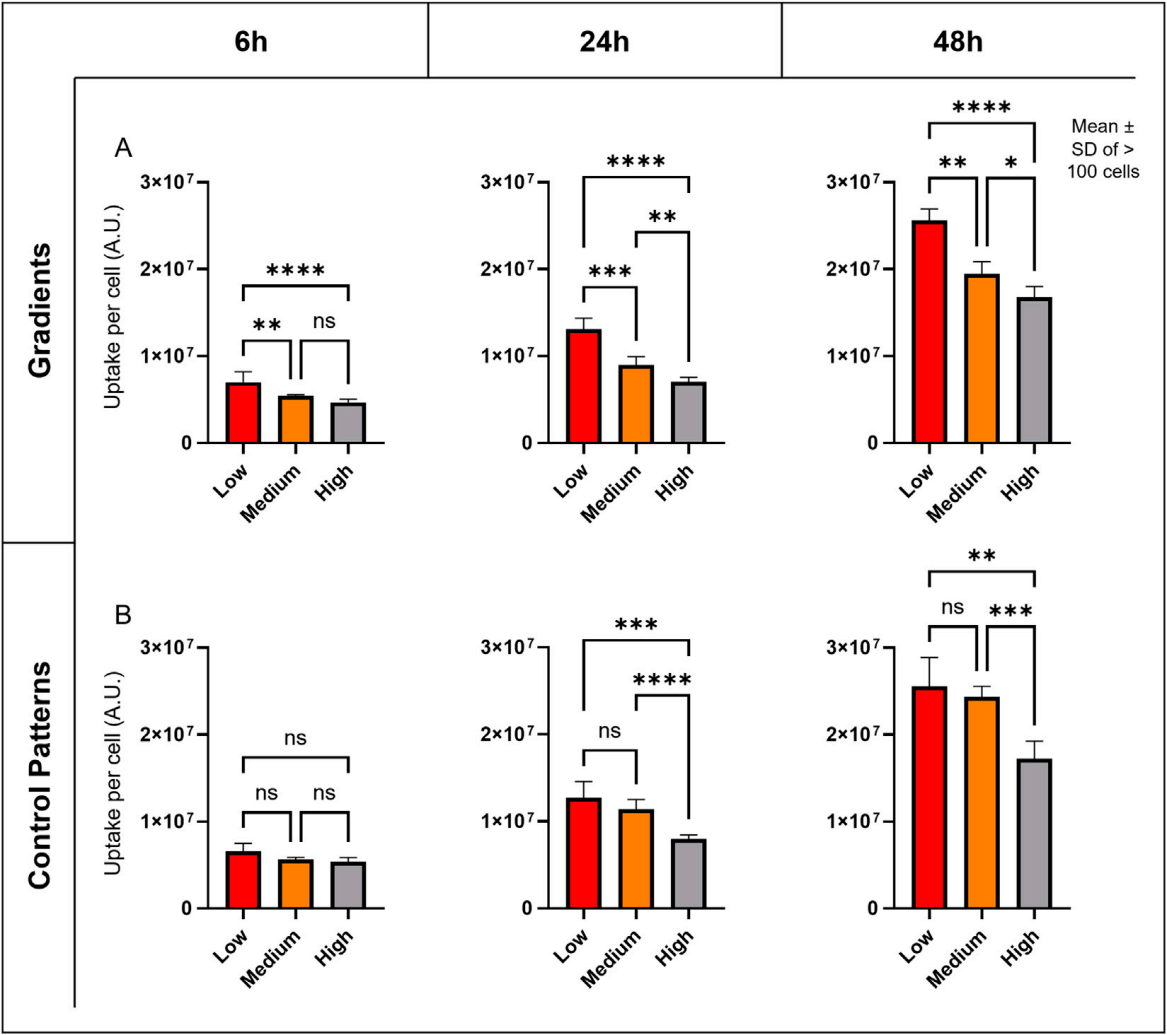
Cell Density’s Impact on NP Uptake. **(A)** Graphs showing the mean and standard deviation (SD) of semi-quantitative NP uptake measurements at different time points in the graded patterns. **(B)** Graphs showing the mean and SD of NP uptake at different time points in the control patterns, with different densities in separate inserts. Signal intensity (A.U.) was quantified from 3D regions of interest (ROIs) derived from 5 image stacks per density zone or pattern. A two-way ANOVA was performed, and variance homogeneity and normal distribution of the ANOVA residuals were verified. Significance annotations reflect the p values: ns = not significant, p < 0.05 (*), p < 0.01 (**), p < 0.001 (***), and p < 0.0001 (****). Across both graded and uniform control patterns, NP uptake increased by approximately 50% from high-density to low-density regions at all time points.

The uptake of ∼112 nm SiO2 NPs (Table 2; Supplementary Figure S2) was analyzed in A549 cells across different density regions using cLSM and 3D image analysis (Figure 3). Quantification of NPs in single cells revealed that cells at low densities exhibited approximately 50% higher NP uptake compared to those at high densities, and this difference remained consistent over the 48 h exposure period (Figure 3A). Similar NP contents were observed in control experiments, where cell density was varied in separate wells (Figure 3B).

**FIGURE 4:**
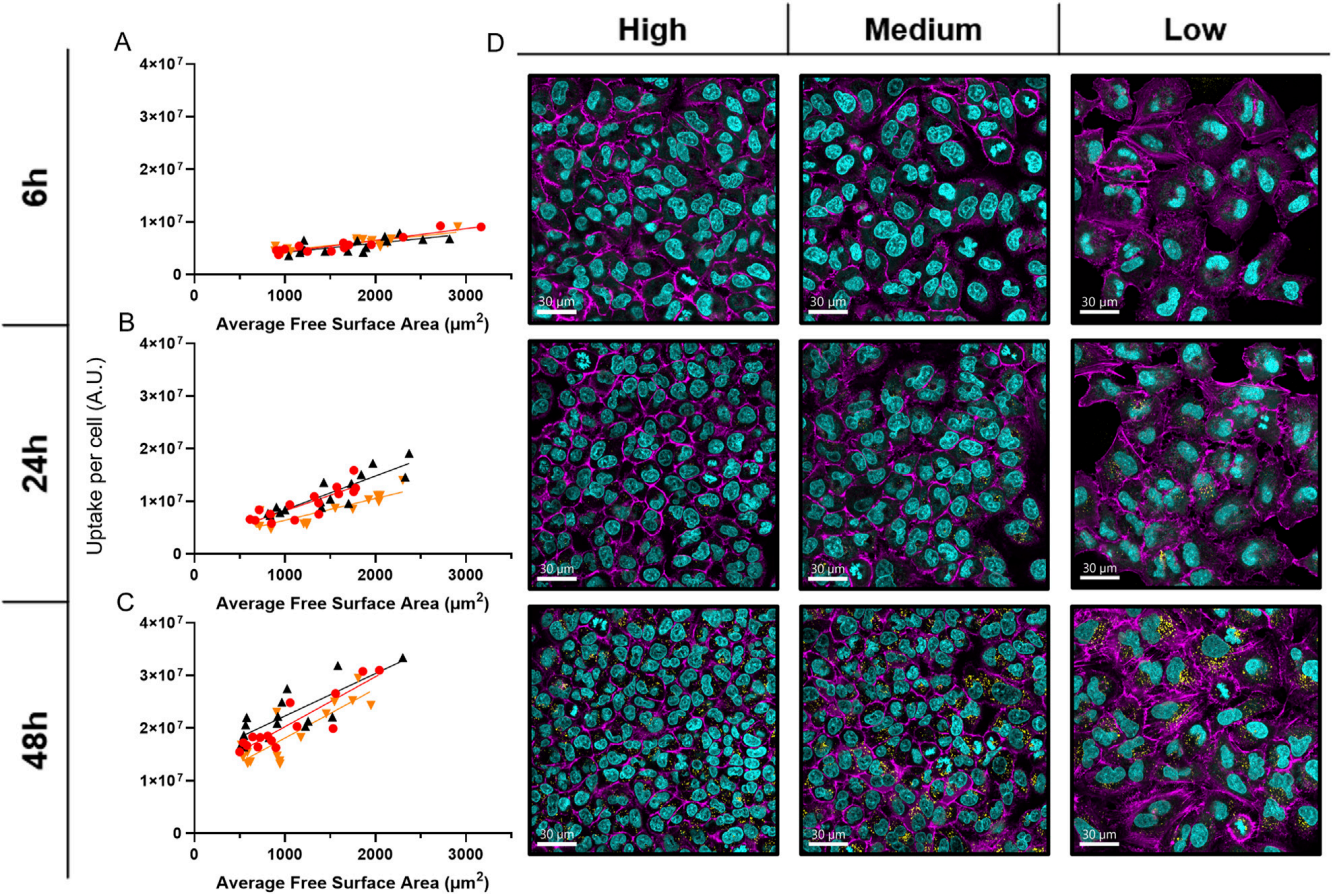
Average Free Surface Area’s Impact on NP uptake. **(A–C)** Graphs showing scatter plots and simple linear regression of NP uptake measurements versus the average free surface area of cells at 6 h A), 24 h B), and 48 h C) in the graded patterns. Different colors represent biological replicates. **(D)** Close-up (63x) cLSM images of the three density zones from representative cell density gradients across all time points, arranged from left to right (high to low density) and top to bottom (6–48 h). These images illustrate differences in cell spreading and crowding over time. Cells were stained with DAPI (cyan) to label nuclei, Alexa Fluor 488 phalloidin (magenta) to visualize F-actin, and internalized NPs were detected via Cy5 labeling (yellow).

Analysis of average free cell surface area and cell volume’s impact on NP uptake showed a strong positive linear correlation at all time points (Figure 4A–C), with Pearson’s correlation coefficients generally above 0.8 and Spearman’s coefficients mostly above 0.7 (Supplementary Figures S6, 7). In brief, larger, more spread cells exhibit higher NP fluorescence, suggesting increased nanoparticle uptake. Indeed, cLSM revealed distinct differences in cell arrangement: cells in high-density regions formed tight, vertically oriented clusters, whereas cells in low-density regions displayed a more spread-out, horizontally arranged morphology (Figure 4D).

Interestingly, cells at higher densities exhibit 30%–50% increased efficiency in NP uptake per unit surface area (µm^2^) compared to cells at lower densities (Supplementary Figure S8). This highlights the significant impact that NP availability has on uptake, as lower-density cells internalize more NPs on average despite being less efficient relative to their surface area.

### 3.4 The impact of cell proliferation on SiO_2_ NP uptake

With all factors considered, one question remains: how much does cell proliferation influence uptake results? To answer this question, the cell proliferation in the control patterns at each density condition had to be estimated.

**FIGURE 5:**
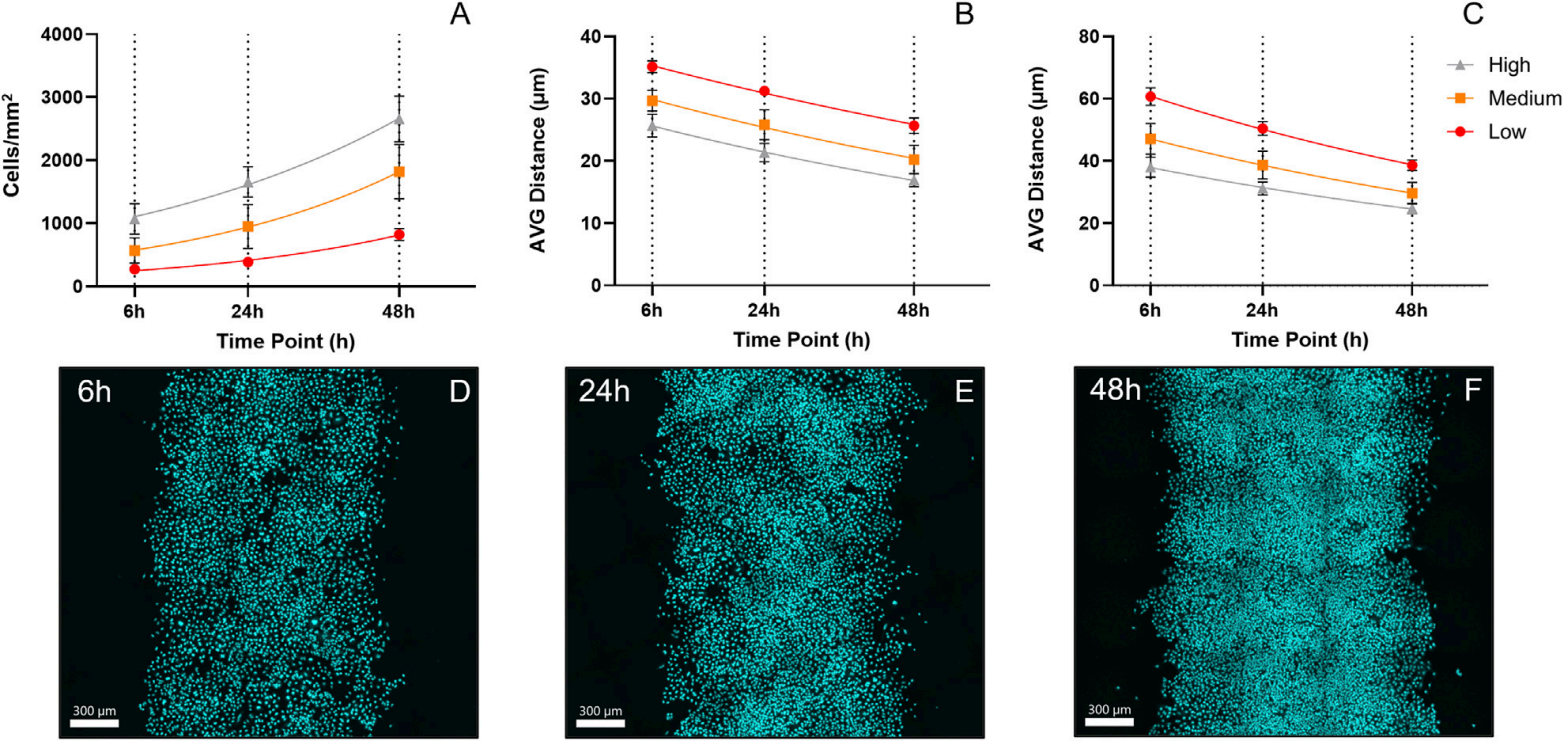
Cell Proliferation and Crowding Dynamics Over 48 h. **(A)** Graph showing cell counting results from control patterns at three different initial densities across the time points of interest, including the exponential fit used to calculate doubling times. **(B)** Graph illustrating the decrease in the average distance to the three closest nuclei. **(C)** Graph showing the decrease in the average distance to the nine closest nuclei. Both metrics were included to capture crowding effects at different scales. The two average distances reflect short- and medium-range crowding within the population. This is particularly relevant at low densities, where nuclei distances can vary greatly due to clustered growth, with examples of tightly packed small clusters remaining isolated **(D–F)** Tile scan (20x) cLSM images from three representative high-density control patterns across all time points (6–48 h, arranged left to right).

Cell proliferation analysis across different densities showed exponential growth patterns over 48 h (Figure 5A). Cell counting revealed doubling times of 25.79 ± 8.66 h in low-density regions, 26.38 ± 8.06 h in medium-density regions, and 33.48 ± 5.35 h in high-density regions, though these differences were not statistically significant. As time progressed, there was a demonstrable decrease in the average distance between the nuclei (Figure 5B, C). The reduction in average nuclear distance over time reflects increasing cell crowding, suggesting that proliferation contributed more to densification at the center of the control patterns rather than driving the outward expansion beyond their initial boundaries.

Confocal microscopy tile scans of control patterns were acquired and used for density estimation. Their visual inspection clearly demonstrated the dynamic increase in cell density and crowding from 6 to 48 h (Figure 5D–F).

## 4 Discussion

### 4.1 The impact of cell density on SiO_2_ NP uptake

The observation of 50% higher NP uptake in low-density regions compared to high-density areas reveals the critical but unrecognized role of cellular spatial organization in NP-cell interactions. Unlike traditional methods (Ashraf et al., 2020; Rennick et al., 2021), our integrated approach provides comprehensive insights by simultaneously examining NP content and cell spatial arrangement within a single experiment. These perspectives are typically assessed independently, with 3D cell surfaces generally being derived rather than directly measured (Shahinuzzaman and Barua, 2020).

The consistency of the density-dependent differences in uptake across all time points suggests fundamental physical constraints rather than temporal cellular adaptations. This also indicates that saturation effects, such as reaching cell content limits or internalization mechanism saturation, did not significantly influence uptake rates, as such effects would typically reduce differences over time by causing uptake in low-density regions to plateau once saturation is reached (Ashraf et al., 2020; Chithrani et al., 2006; Lunov et al., 2011).

Cell density is inherently connected to cell spreading and has been shown, particularly in mesenchymal stem cells, to be a more critical determinant of cell morphology and proliferation than substrate stiffness (Venugopal et al., 2018). Therefore, the impact of the average free cell surface area and cell volume on uptake was investigated. A strong positive linear correlation was found between cell surface area, cell volume, and NP uptake, with correlation coefficients (Pearson’s r and Spearman’s ρ) generally well above 0.7, with a minimum of 0.6 (Supplementary Figures S6, 7). This correlation between average accessible cell surface area and NP uptake provides quantitative evidence that spatial accessibility of the outer cell membrane significantly influences cellular interactions with NPs. This effect, which explains 50%–80% of uptake variability through surface area availability, aligns with previous studies showing relationships between cell size, area, and NP uptake (Shahinuzzaman and Barua, 2020; Khetan et al., 2019; Behzadi et al., 2017; Yang et al., 2023).

However, the remaining 20%–50% of variability may stem from additional factors. These include density-dependent cellular behaviors beyond surface area influence, such as variations in receptor expression or membrane dynamics (Zhang et al., 2021; Garcia Romeu et al., 2021; Steinkühler et al., 2019; Kavaliauskiene et al., 2014). In future studies, immunostaining of receptors or membrane-bound proteins linked with specific endocytic pathways (transferrin receptor, caveolin-1) (Bannunah et al., 2024; Feng et al., 2020) and the use of lipid-specific fluorescent probes (filipin, Laurdan) (Wilhelm et al., 2019; Levitan, 2021) could be applied to assess density-dependent variations in receptor expression and membrane composition.

Additionally, cell over-growth at higher densities may affect average free surface area and NP uptake quantification (Supplementary Figure S5) by reducing surface accessibility and introducing depth-related detection biases; however, z-stacks were acquired while avoiding regions with significant multilayer growth to minimize this bias. Another possible factor could be cells at lower densities moving more freely, increasing NP availability. Lastly, the limitations in cell segmentation, counting accuracy, and surface/volume quantification methods could introduce measurement biases and lead to slight over- or underestimations of these parameters. Further optimization of the image acquisition and image processing approach will reduce the effect of these limitations and enhance the accuracy of the results.

Further evidence of the fundamental role of cell spreading and available free surface area in NP uptake was observed when analyzing the impact of cell density on uptake efficiency per µm^2^ (Supplementary Figure S8). Cells at higher densities demonstrated 30%–50% greater efficiency in NP uptake per unit area compared to lower-density cells, suggesting that while lower-density cells uptake more NPs on average, they are less efficient relative to their surface area. In this study, the authors prioritized analyzing NP uptake on a per-cell basis to reflect the primary interest in potential effects at the single-cell level. However, expressing uptake efficiency relative to surface area would be more relevant to investigating tissue-level functions, such as overall uptake capacity and barrier properties.

Our 3D analysis approach provides advantages over traditional 2D assessments (Khetan et al., 2019; Yang et al., 2023; Rees et al., 2019), by directly considering the entire accessible cell surface rather than just the projected area or simulated 3D estimations. This also differs from flow cytometry, which typically estimates cell size from detached, rounded cells (Shin et al., 2020; Adan et al., 2017). While our approach does not yet differentiate between apical and basal surfaces, it more accurately represents NP interactions under real testing conditions by excluding surfaces between contacting cells. These findings enhance our understanding of how cellular organization influences NP-cell interactions at different cell densities.

The A549 cell line was selected for its widespread use in studies investigating nanoparticle–cell interactions. Furthermore, the bioprinting process did not adversely affect cell viability, making A549 cells particularly suitable for assessing how variations in cell density influence nanoparticle uptake by modulating cell spreading and the resulting exposed surface area.

The selection of the 20 μg/mL SiO_2_ NP dispersion is based on previous studies showing SiO_2_ NPs’ low toxicity at this concentration (Kim et al., 2017; Yu et al., 2009; Mares-García et al., 2021) while allowing sufficient particle internalization (Moreno-Echeverri et al., 2022; Lee et al., 2022). Moreover, the SiO_2_ NPs were suspended in the growth medium containing 10% FBS, which has been reported to reduce their cytotoxicity due to protein corona formation (Sun et al., 2018; Mortensen et al., 2013). This agrees with our study and no significant impact on cell viability or cell metabolism was observed. For all experiments and conditions, the same particle concentration was used. For cells cultured directly in 96-well plates, a volume of 100 µL per well was used to meet the viability assay volume requirement. This exposure corresponded to a nanoparticle surface dose of approximately 6 μg/cm^2^, calculated by normalizing the total NP amount in the exposure volume to the well area. The surface dose was chosen to be comparable to that received by cells printed on inserts, where 1 mL of a 20 μg/mL dispersion corresponds to approximately 5 μg/cm^2^. Similarly, cells cultured in 24-well plates to study the NP’s effect on proliferation were exposed to 500 µL of the dispersion, achieving a comparable surface dose of around 5 μg/cm^2^.

### 4.2 The impact of cell proliferation on SiO_2_ NP uptake

Initial cell seeding density has been recognized as a critical factor influencing cell proliferation for primary cells (Heng et al., 2011; Kim et al., 2016) as well as cell lines. For adenocarcinoma epithelial cell lines, studies report contrasting findings, with higher densities being associated with either slower (Browning et al., 2018), faster (Jin et al., 2017; Jin et al., 2016), or unaffected (Browning et al., 2020) proliferation rates.

Our findings reveal variations in doubling times across density regions, though not statistically significant. The consistent ratio of average NP content between low and high-density regions (1.5x) over time indicates that proliferation-related NP dilution (Kim et al., 2012; Bourquin et al., 2019; Summers, 2012) had minimal influence on the system, and uptake rates remained stable. Since higher-density regions typically experience slower proliferation, they should undergo less NP dilution over time compared to low-density regions. If proliferation-driven dilution were a dominant factor, we would expect the NP content in low-density regions to decline relative to high-density regions over time, thereby decreasing the low-to-high NP content ratio.

These variations in doubling times observed in the printed control patterns contrast with proliferation results in standard culture conditions, where doubling times showed greater consistency across densities. The proliferation rates in standard cultures in 24-well plates (Supplementary Figure S1) remained consistent across all densities and time points, ranging between 18 and 20 h in doubling time for both exposed and control cells. Comparing these with the pattern-based results, where doubling times varied from 25.79 to 33.48 h, highlights the importance of culture conditions in cellular behavior. The faster proliferation in well plates compared to printed control patterns could be attributed to the higher perceived cell density (Enrico Bena et al., 2021; Nelson and Chen, 2002; Johnson et al., 2019) in the former situation, with cells typically accumulating at the center of the wells after pipetting (Reynolds et al., 2018; Glaubitz et al., 2023). Cell counts obtained using the automated cell counter, however, may have been affected at early time points and at lower initial seeding densities, where concentrations were outside the instrument’s optimal detection range. Additionally, cell concentrations that were derived from the imaged control patterns may have been underestimated at higher densities. Nuclear morphology and dimensions were observed to change across different cell densities, decreasing in size at increasing crowding conditions. This behavior is consistent with known effects of varying crowding and cell spreading on nuclear structure, as reported in several cell lines and primary cells (Cantwell and Dey, 2022; Edens et al., 2013; Bermudez et al., 2024; De Corato and Gomez-Benito, 2024). This could have impacted counting accuracy at increasing crowding conditions and could partially explain why a variation in proliferation rate, though not significant, was observed at different densities in the control patterns, while no difference was found for cells cultured in the well plates.

As a cancer cell line, A549 exhibits reduced contact inhibition compared to primary cells, constituting a limitation of the model. The experimental time frame was carefully selected to ensure that A549 cells largely maintained monolayer growth, with minimal multilayer formation. Future studies incorporating primary cells would help validate and extend these findings by providing additional information on the impact of proliferation on NP uptake under conditions that more accurately represent a healthy epithelium, where cell density is physiologically regulated.

In this study, A549 cells are cultured and exposed under growing conditions. This differs from the in-situ situation in the healthy lung, where the cells are in a differentiated state instead of a proliferative one (Juul et al., 2020; Spencer and Shorter, 1962; Guillot et al., 2013). This was deemed necessary in order to ensure robust and sensitive responses in viability assays, which rely on active metabolism. Moreover, inducing a non-proliferative state *in-vitro* would require methods that would additionally affect the results, like serum starvation or the use of chemical inhibitors. Lastly, as A549 cells are derived from alveolar Type II cells, which serve as facultative stem cells capable of proliferating and differentiating during lung repair, expanding conditions remain relevant particularly when modeling tissue regeneration in pathological contexts (Barkauskas et al., 2013; Ruaro et al., 2021).

### 4.3 Optimized fabrication of cell gradients

The choice of using a linear gradient rather than multiple discrete uniform-density patterns in this experimental setup was driven by its potential to enable continuous exploration of a virtually unlimited range of cell densities within a single run. An “area gradient” created by juxtaposing parallel uniform patterns was also considered but not selected due to the increased difficulty in controlling printing result consistency, as well as in recognizing the density zones of interest during microscopic analysis.

Although effective, existing methods to fabricate cell density gradients have significant limitations. Sedimentation-based and directed cell migration approaches suffer from lengthy preparation times, parameter control difficulties, and poor reproducibility (Liu et al., 2013; Havenhill and Ghosh, 2024; Frick et al., 2018; Fortunato and Sunyer, 2022; Wu et al., 2014), while microfluidic and 3D printing platforms employing microfluidic chips for mixing and gradient formation require substantial resources and specialized expertise (Mehling and Tay, 2014; Chiu et al., 2000; Li et al., 2015a; Kuzucu et al., 2021; Idaszek et al., 2019). The successful establishment of a straightforward and reproducible method to prepare cell density gradients through the use of drop-on-demand bioprinting technology provides a novel platform for investigating density-dependent cellular interactions with NPs in one dish.

This method enables accurate and controlled gradient formation with reduced preparation time, without requiring specialized expertise in microfluidics or advanced 3D printing. While our results demonstrate reproducibility across independent runs, further investigation and stricter quantitative analysis of the method’s variability will be conducted to strengthen this conclusion.

The development of stable and uniform cell gradients presented significant technical challenges, particularly in maintaining the shape stability of the deposited cell-laden growth medium. In this regard, better droplet stability after bioprinting on PET hanging inserts with 3 µm pores was observed compared to inserts with smaller pore sizes and other substrates, including those functionalized with hydrophilic agents such as poly-L-lysine (PLL), APTES, and collagen type I. However, a detailed comparison of substrate performance is beyond the scope of this study and no quantitative data are presented. Implementing a pre-layer of medium before cell deposition significantly improved gradient and control pattern reproducibility across different cell passages and printing processes, by facilitating controlled droplet merging.

This optimized approach enables reliable investigation of cell density effects on NP uptake in a controlled environment, advancing our understanding of density-dependent cell behavior. Additionally, these graded patterns represent a strong framework to investigate further the impact of cellular organization and spatial arrangement on NP-cell interactions and cell physiology, with important implications for drug delivery and nanosafety evaluations.

## 5 Conclusion

This study highlights the significant impact that cell density exerts on NP uptake in the tested epithelial cell line model, with cells growing in lower-density regions demonstrating around 50% higher uptake than those in high-density areas up to 48 h after exposure. This effect strongly correlates with the average available cell surface area, indicating its critical impact on NP-cell interactions. While variations in cell proliferation rates between densities were observed in the printed control patterns, these differences were not statistically significant and did not contribute to the observed differences. This indicates that factors like cell spreading, and average cell volume have a more substantial impact under the tested conditions.

Using the A549 epithelial cell line and amorphous SiO_2_ NPs (∼112 nm) provided a controlled system to investigate the relationship between cell density and NP uptake. Future studies should expand to primary cells, which exhibit increased contact inhibition (Grimes and Fletcher, 2020; Pavel et al., 2018), and consider additional NP properties like size, shape, and surface chemistry (Öztürk et al., 2024; Li et al., 2015b; He et al., 2010; Kettler et al., 2014; Foroozandeh and Aziz, 2018; Sousa De Almeida et al., 2021). Furthermore, investigating whether endocytic mechanisms themselves are density-dependent would be valuable, given reported variations in membrane properties and protein expression under different density conditions (Trajkovic et al., 2019; Steinkühler et al., 2019; Kavaliauskiene et al., 2014; Nussinov et al., 2021; Murate et al., 2023; Shim et al., 2020), and the differences in uptake efficiency per µm^2^ (30%–50%) observed in this study at different crowding conditions.

Based on these results, this study highlights the importance of investigating density-dependent nanoparticle uptake in cell types that are particularly involved in pathological contexts. In fact, inflammation and tissue repair are driving processes impacting the degree of cell density variability in such tissues. The lung epithelium is an especially relevant example because of its constant exposure to airborne particulates (Portugal et al., 2024), its susceptibility to chronic inflammatory diseases (Duncan, 2016; Barnes et al., 2015), and its role as a major route of administration for both nanomedicines and conventional drugs (Wu L. et al., 2020; Videira et al., 2020). On the other hand, modeling healthy tissues and conditions where *in-vivo* cell density is less variable requires careful consideration of the experimental cell density of choice, as the reliability and translatability of the results are directly impacted.

To conclude, this work proposes an approach to systematically investigate density-dependent effects with the opportunity to adapt the density range of interest depending on the research question. Moreover, it can be applied to mimic pathological conditions characterized by high spatial variability in cell density, providing insights into how such heterogeneity influences nanoparticle interactions.

The discussed findings increase our understanding of how cellular spatial organization influences NP-cell interactions and highlight the importance of considering the cell density factor in nanosafety evaluations and drug delivery design. Future works should incorporate more complex biological models to advance this understanding and its applications.

## Data Availability

The datasets presented in this study can be found in online repositories. The names of the repository/repositories and accession number(s) can be found below: https://doi.org/10.5281/zenodo.14936452.
