# Supplementary material for: The impact of cell density variations on nanoparticle uptake across bioprinted A549 gradients
Source: Front Bioeng Biotechnol. 2025 Apr 30;13:1584635. doi: 10.3389/fbioe.2025.1584635 (PMC12075422; doi:10.3389/fbioe.2025.1584635)
Supplement: Supplementary file 1 [file DataSheet1.docx]

**The Impact of Cell Density Variations on Nanoparticle Uptake Across Bioprinted A549 Gradients**

Luigi Di Stolfo^1^, Wang Sik Lee^1^, Dimitri Vanhecke^1^, Sandor Balog^1^, Patricia Taladriz-Blanco^1^, Alke Petri-Fink^1,2^ and Barbara Rothen-Rutishauser^1*^

^1^Adolphe Merkle Institute and National Center of Competence in Research Bio-Inspired Materials, University of Fribourg, Switzerland, Chemin des Verdiers 4, Fribourg, Switzerland.

^2^Department of Chemistry, University of Fribourg, Chemin du Musée 9, Fribourg,

Switzerland

*Corresponding author:

Prof. Barbara Rothen-Rutishauser

[barbara.rothen@unifr.ch](mailto:barbara.rothen@unifr.ch)

##
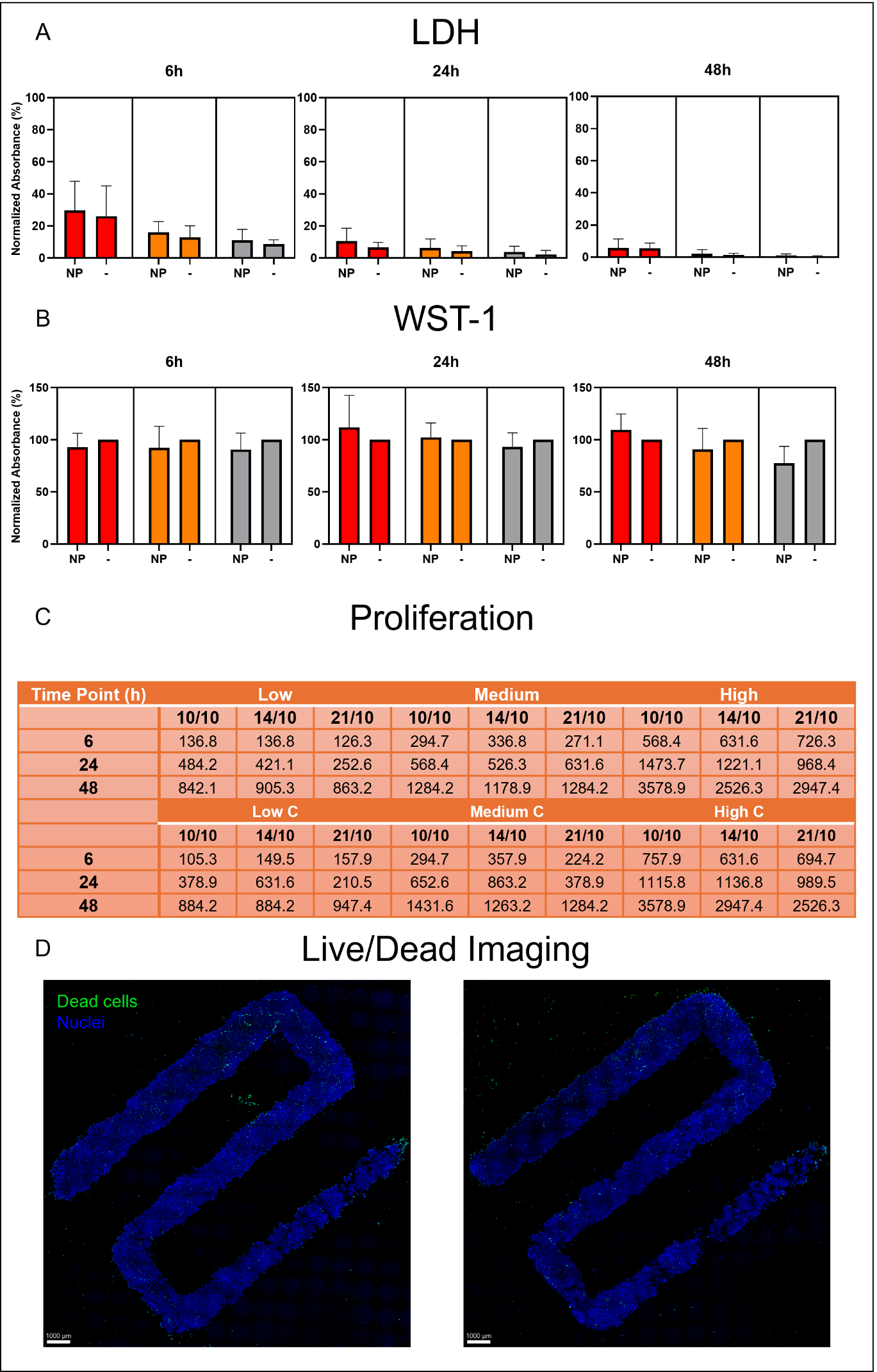


## Figure S1

Non-Toxic Interaction Overview. A) Normalized LDH release results for cells seeded at different densities following exposure to F-SiO_2_ NPs across all time points. Cells at lower densities exhibited proportionally higher LDH release in both exposed and control groups. This may be due to increased LDH degradation over time in Triton-X-treated samples, an effect that was more apparent at lower densities. B) WST-1 assay results assessing metabolic activity of cells at all time points after exposure to the SiO_2_ NPs. No significant changes in metabolic activity were observed, except for a slight, non-statistically significant reduction in high-density cultures at 48 hours. C) Comparison of A549 cell proliferation rates in the presence and absence of SiO_2_ NP exposure over 48 hours. The results are presented as the total cell count from the automatic counter divided by the well area. No substantial differences in proliferation were detected, indicating no significant impact of nanoparticle exposure on cell growth under the conditions tested. D) cLSM 10x magnification tile scan images of gradients stained with Live/Dead imaging kit (dead cells in green; nuclei in blue), showing minimal cell death after printing and 48h NP exposure.


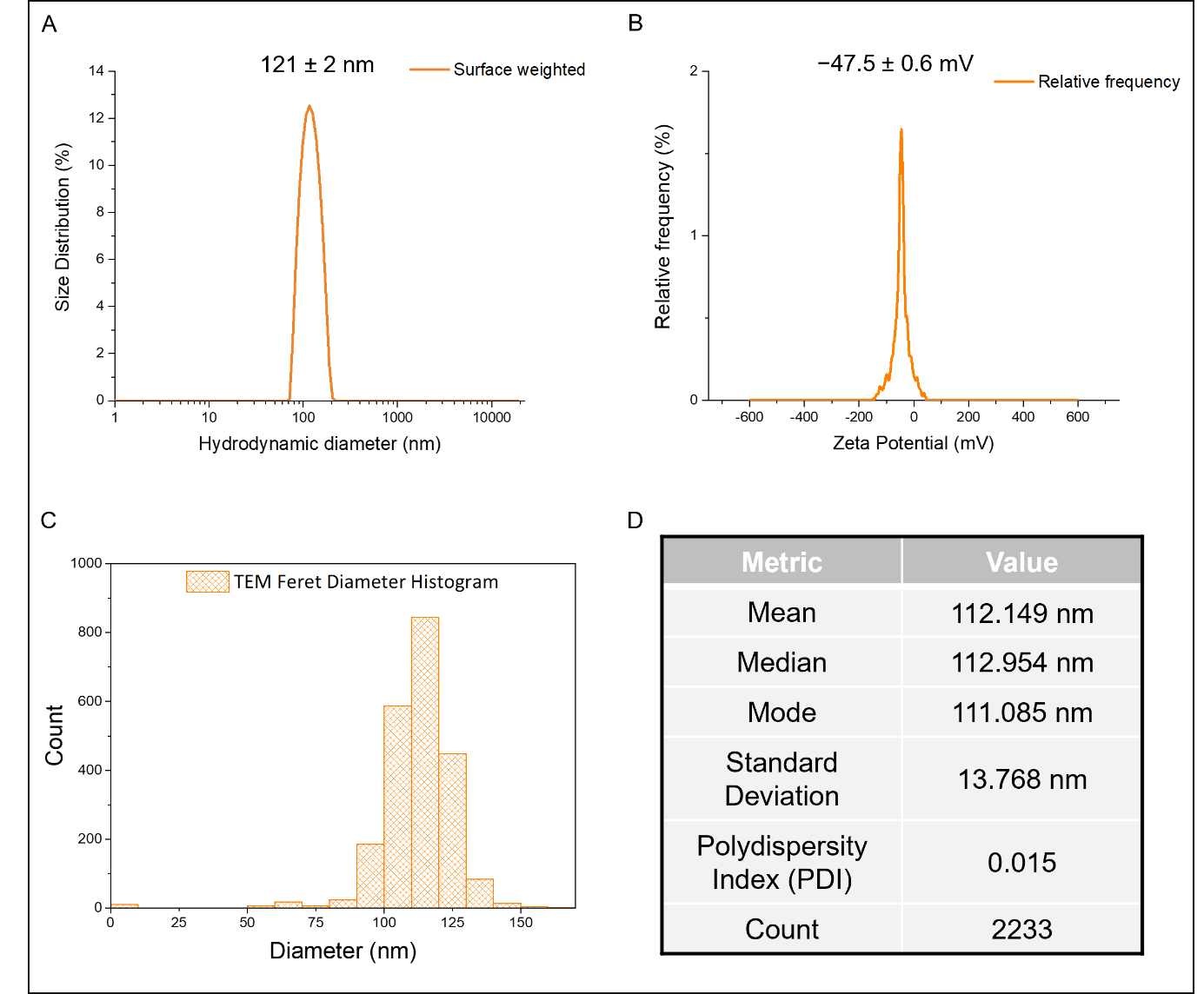


## Figure S2

NPs characterization. A) Surface-weighted size distribution graph showing the hydrodynamic diameter of the NPs (20 µg/mL) in Milli-Q water, measured at 25 °C using dynamic light scattering (DLS). B) Histogram of the zeta potential distribution of the SiO_2_ NPs (20 µg/mL) at 25 °C in Milli-Q water, illustrating relative frequency. C) Histogram of the Feret diameters of the SiO_2_ NPs calculated from transmission electron microscopy (TEM) images. D) A summary table presenting key metrics of the SiO_2_ NPs obtained from TEM analysis.


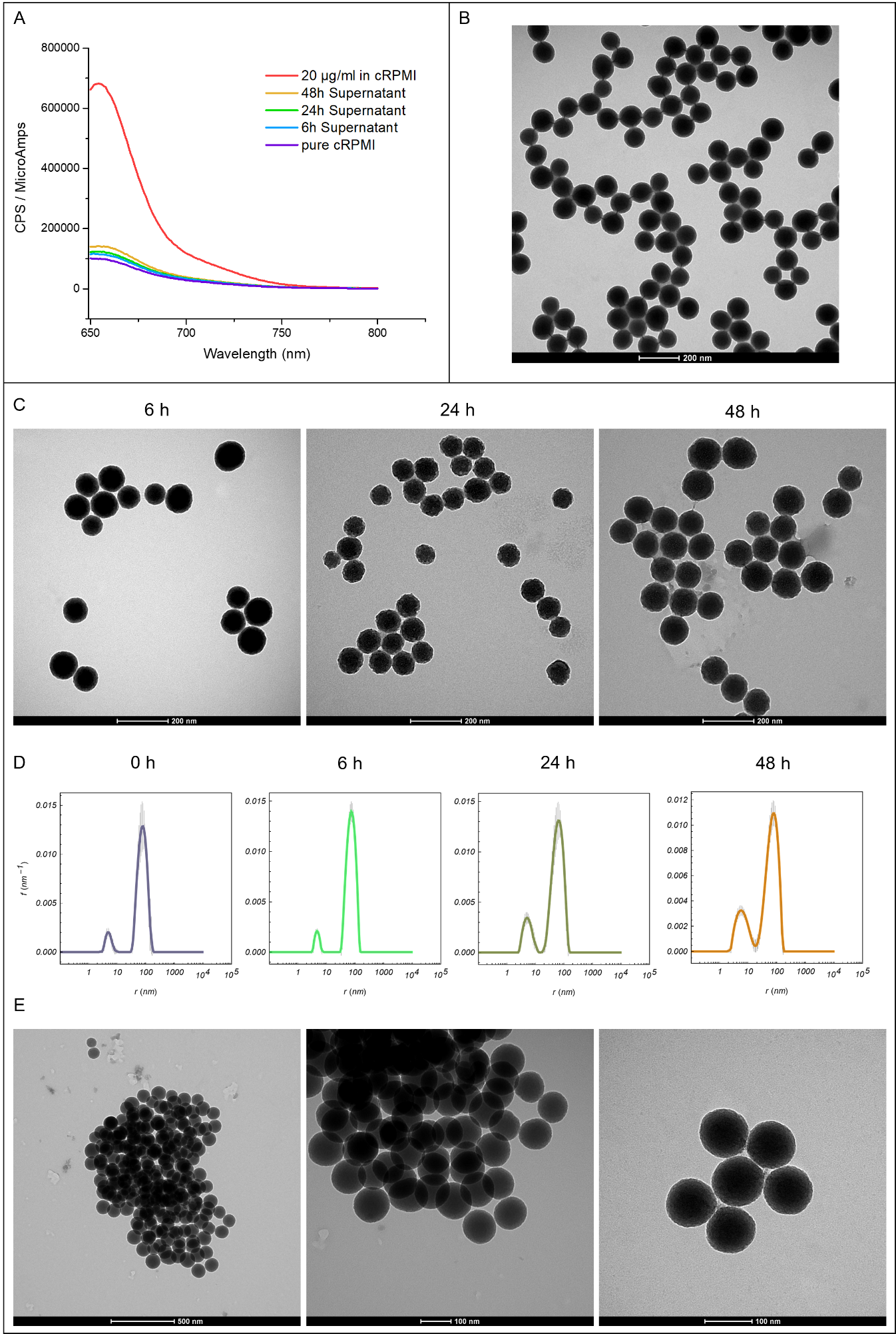


## Figure S3

NPs stability and label leakage. A) Fluorimetric results of the growth medium (Phenol Red-Free cRPMI) after 6, 24, and 48 hours of NP incubation, compared to pure medium and a 20 µg/mL F-SiO_2_ NP dispersion. B) TEM micrograph of the SiO_2_ NPs from the stock solution prepared in Milli-Q water. C) TEM micrographs showing the morphological evolution of the SiO_2_ NPs after 6, 24, and 48 hours of incubation in cRPMI. D) DLS-derived hydrodynamic diameter distributions of the SiO_2_ NPs, presented sequentially from left to right for 0, 6, 24, and 48 hours of incubation in the medium. E) TEM micrographs showing the SiO_2_ NP stock in Milli-Q water after 9 days from the last NP uptake experiment, confirming the stability of the NPs during storage throughout the experimental period.


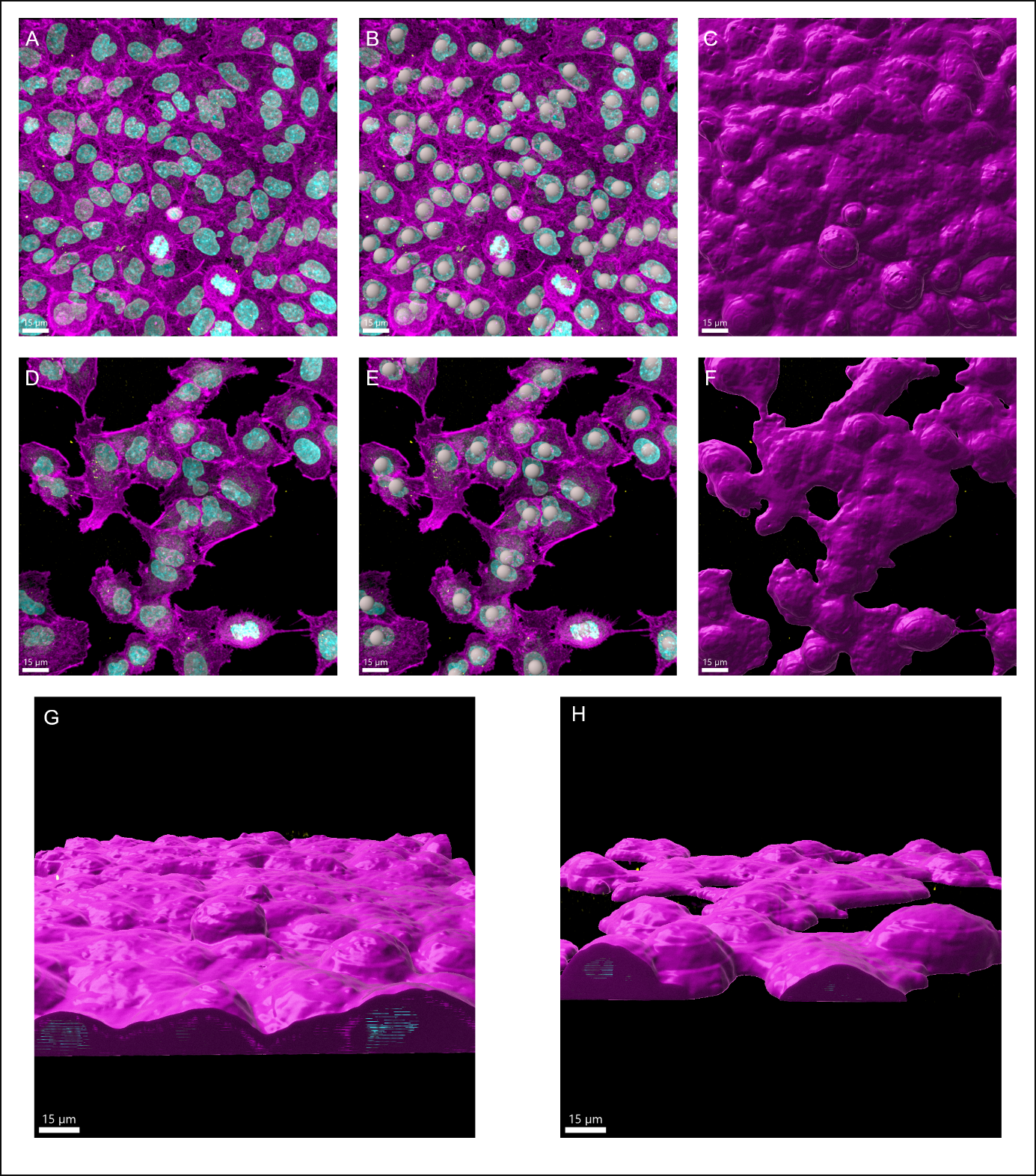


## Figure S4

3D cell visualization and analysis. A) Top-down view of a 3D reconstruction from a representative stack in the high-density zone of the gradient, with nuclei in cyan, cytoskeleton (actin) in magenta, and NPs in yellow. B) Cell nuclei quantification method applied to the high-density stack. C) Top-down visualization of the 3D region of interest (ROI) used to estimate cell dimensions and NP uptake in the high-density stack. D) Top-down view of a 3D reconstruction from a representative stack in the low-density zone. E) Cell nuclei quantification method applied to the low-density stack. F) Top-down visualization of the 3D ROI from the low-density stack. G) Side view of the 3D ROI created from the high-density stack. (H) Side view of the 3D ROI created from the low-density stack.


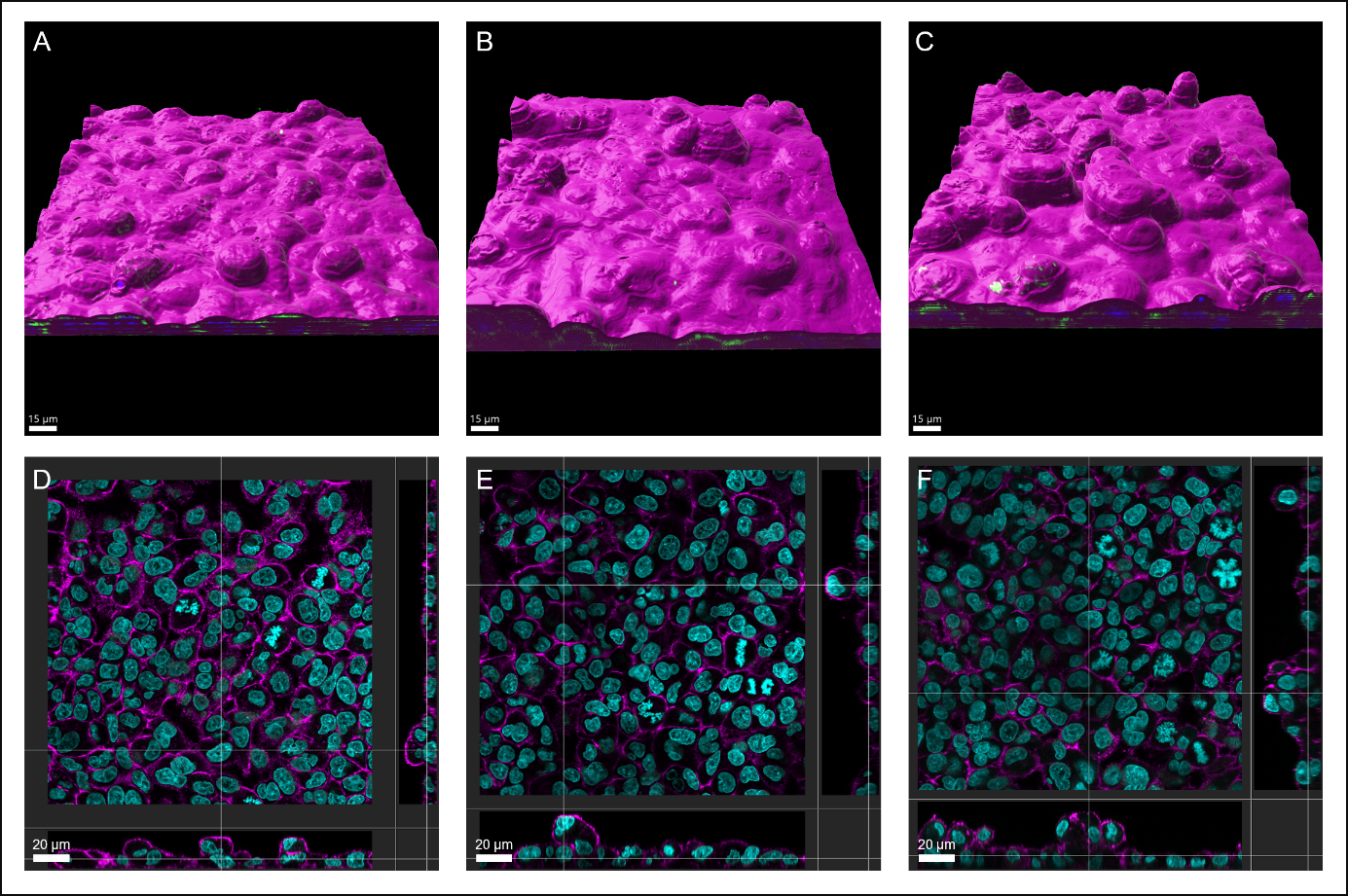


## Figure S5

Multilayer cell growth. A-C) Side views of 3D ROIs created from example stacks showing increasing cell growth on top of the first monolayer. D-F) Orthogonal views of the same stacks showing cells growing on top of the first layer. Multilayer cell growth was observed only in high-density zones for the 24 and 48h exposure time point.


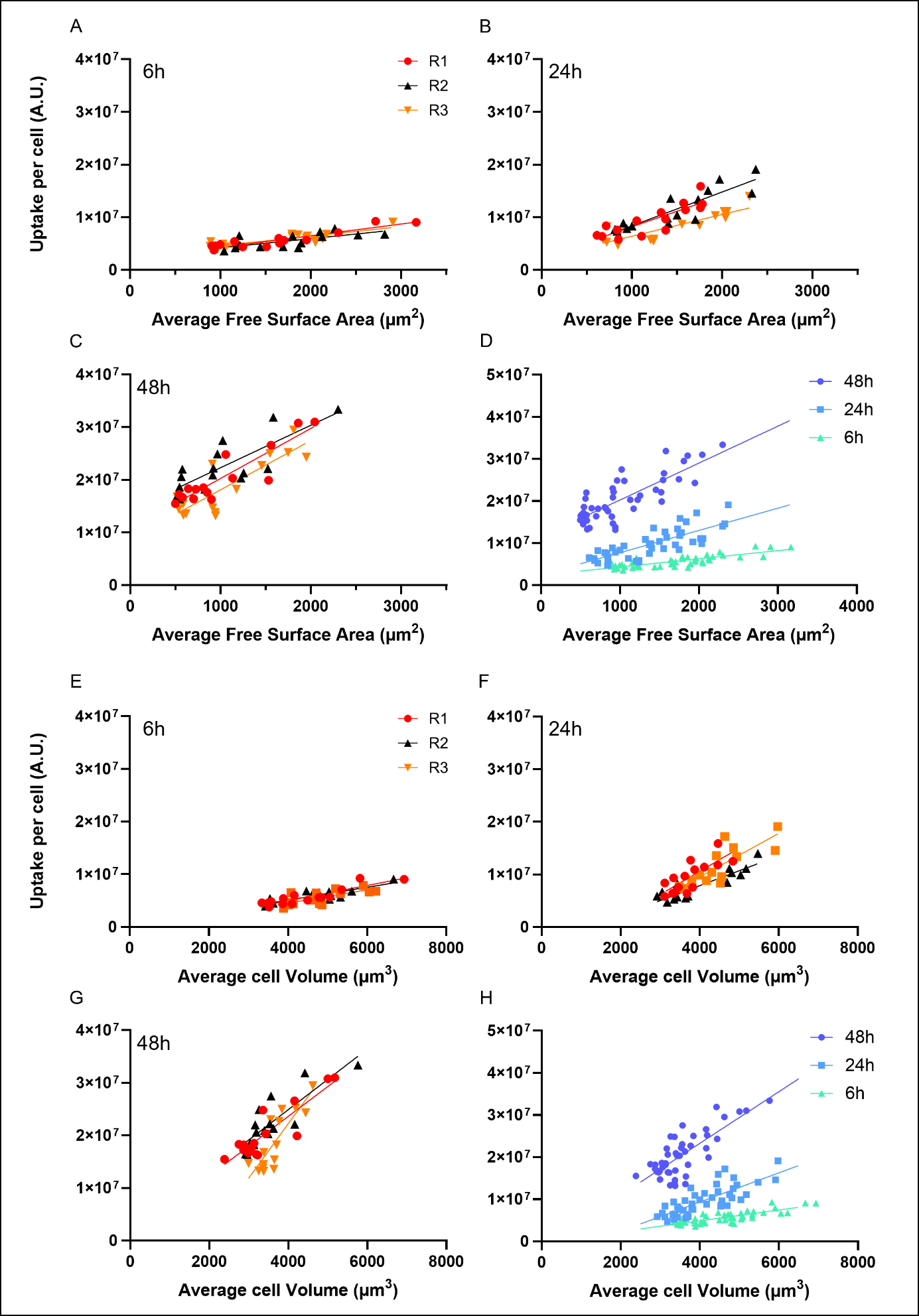


## Figure S6

Cell surface area and volume vs NP uptake. A–C) Scatter plots with linear regression curves representing the relationship between the average free surface area and NP uptake per cell for three biological replicates (R1, R2, R3) at 6, 24, and 48 hours, respectively. D) Scatter plot with linear regression curves showing the relationship between average free surface area and NP uptake per cell, combining biological replicates for each time point (6, 24, and 48 hours) to enable direct comparison across time. E–G) Scatter plots showing the linear correlation between average cell volume and NP uptake per cell, based on data from three biological replicates at all time points. H) Scatter plot with linear regression curves showing the correlation between average cell volume and NP uptake per cell, with biological replicates combined per time point to facilitate direct temporal comparisons.


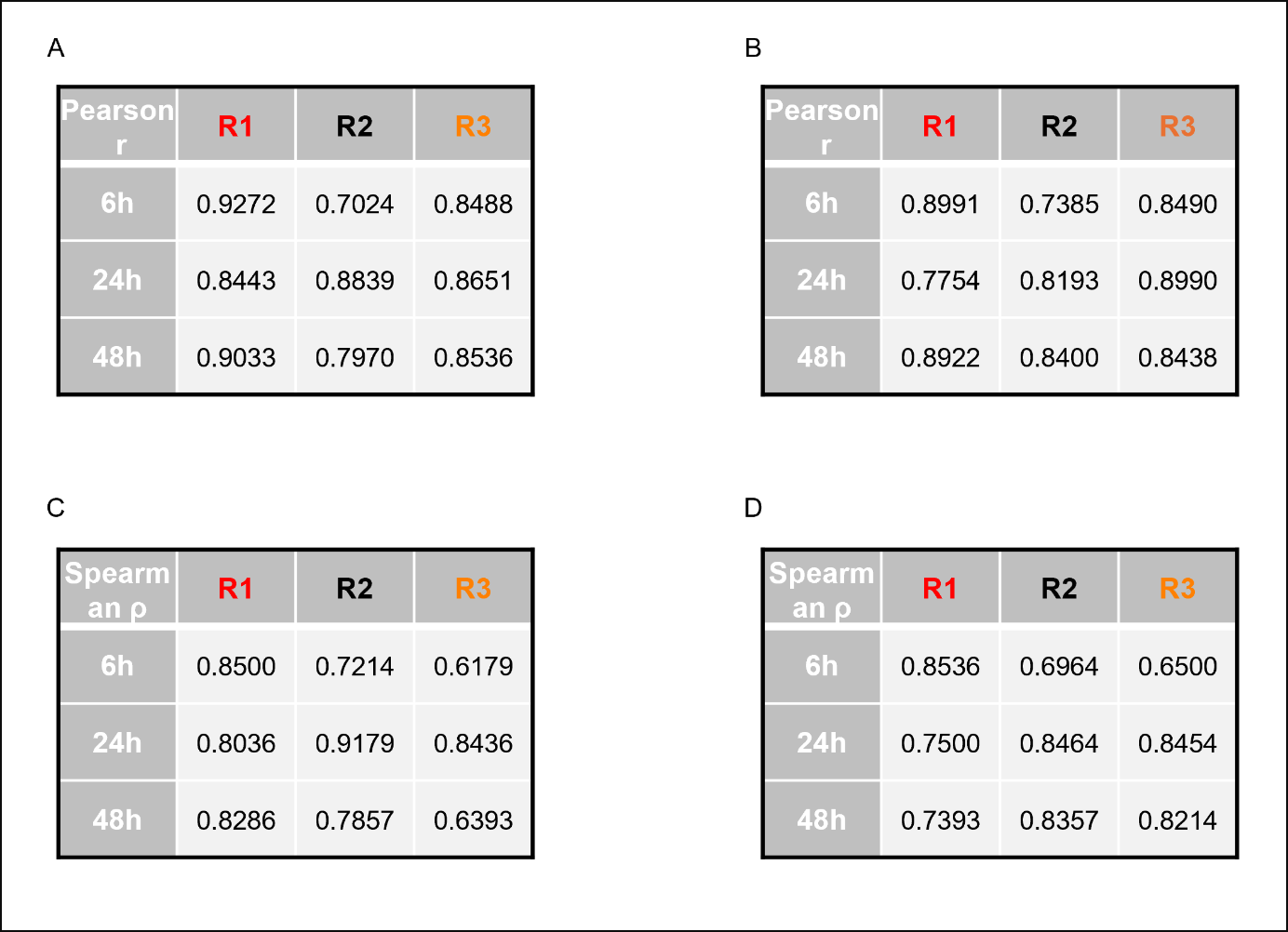


## Figure S7

Cell surface area and volume vs. NP uptake – Comparison of Pearson and Spearman’s correlations: A) Summary table displaying the Pearson correlation coefficients (r) for each biological replicate (R1, R2, R3) across the three time points. B) Summary table of Pearson’s r for NP uptake versus average cell volume for each biological replicate (R1, R2, R3) across three time points. C) Summary table of Spearman’s ρ for NP uptake versus average free surface area pooled across all replicates and time points. D) Summary table of Spearman’s ρ for NP uptake versus average cell volume pooled across all replicates and time points.


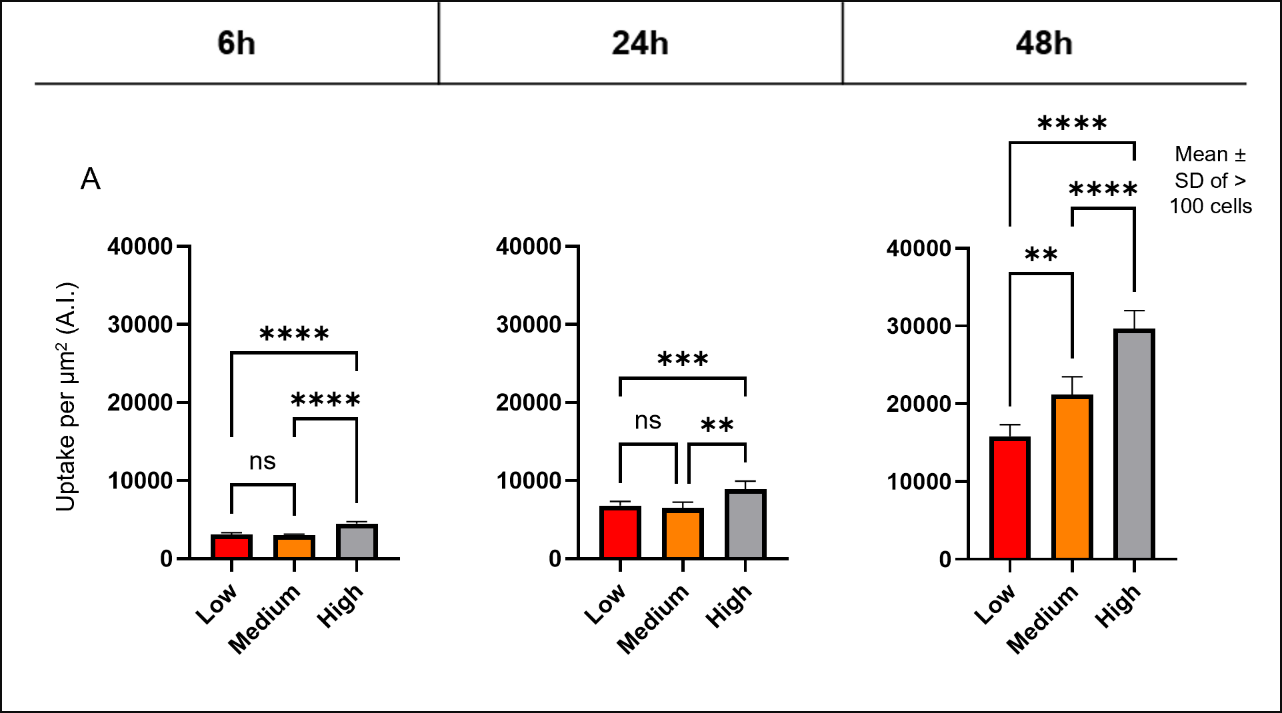


## Figure S8

Cell density’s impact on NP uptake per µm^2^. A) Graphs showing the mean and standard deviation (SD) of semi-quantitative NP uptake measurements, normalized on the cell surface area, at different time points in the graded patterns. A Mixed-Effect Model statistical analysis was performed, given the variance heterogeneity of the data. Significance annotations reflect the p-values. Across both graded and uniform patterns, NP uptake efficiency per µm^2^ increased by approximately 30-50% from low-density to high-density regions at all time points.
